# Increases in adipose tissue and muscle function are longitudinally associated with better quality of life in colorectal cancer survivors

**DOI:** 10.1038/s41598-021-91709-y

**Published:** 2021-06-14

**Authors:** Marlou-Floor Kenkhuis, Eline H. van Roekel, Janna L. Koole, José J. L. Breedveld-Peters, Stéphanie O. Breukink, Maryska L. G. Janssen-Heijnen, Eric T. P. Keulen, Fränzel J. B. van Duijnhoven, Floortje Mols, Matty P. Weijenberg, Martijn J. L. Bours

**Affiliations:** 1 Department of Epidemiology, GROW School for Oncology and Developmental Biology, Maastricht University, P.O. BOX 616, 6200 MD Maastricht, The Netherlands; 2 Department of Surgery, GROW School for Oncology and Developmental Biology, Maastricht University Medical Centre+, NUTRIM School of Nutrition and Translational Research in Metabolism, Maastricht, The Netherlands; 3 Department of Clinical Epidemiology, Viecuri Medical Center, Venlo, The Netherlands; 4 Department of Internal Medicine and Gastroenterology, Zuyderland Medical Centre, Sittard-Geleen, The Netherlands; 5 Department of Human Nutrition and Health, Wageningen University and Research, Wageningen, The Netherlands; 6 Center of Research on Psychological and Somatic Disorders (CoRPS), Department of Medical and Clinical Psychology, Tilburg University, Tilburg, The Netherlands

**Keywords:** Cancer, Cancer epidemiology, Gastrointestinal cancer

## Abstract

Colorectal cancer (CRC) survivors need evidence-based guidelines pertaining to post-treatment body composition, which could benefit health-related quality of life (HRQoL). We aimed to describe the course of several body composition measures, and to assess longitudinal associations of these measures with HRQoL, fatigue and chemotherapy-induced peripheral neuropathy (CIPN). In a prospective cohort among stage I–III CRC survivors (n = 459), five repeated home visits from diagnosis up to 24 months post-treatment were executed. Body mass index (BMI), waist circumference and fat percentage were assessed as measures of adiposity, and muscle arm circumference and handgrip strength as measures of muscle mass and function. We applied linear mixed-models to describe changes in body composition over time and to analyze overall longitudinal associations. Of included participants, 44% was overweight and 31% was obese at diagnosis. All body composition measures followed similar trends, decreasing from diagnosis to 6 weeks and then increasing up to 24 months post-treatment. In confounder-adjusted mixed models, increases in adipose tissue and muscle function were longitudinally associated with better HRQoL and less fatigue, regardless of pre-treatment body composition. With regards to improving HRQoL, decreasing fatigue and CIPN, clinical practice should also focus on restoring body tissues after CRC treatment.

**Trial registration:** NTR7099.

## Introduction

Early detection, improved treatments, and population ageing are causing an increasing number of colorectal cancer (CRC) survivors worldwide^1–3^. Two commonly reported long-term side effects of CRC and/or its treatment include fatigue and chemotherapy-induced peripheral neuropathy (CIPN), which both severely affect health-related quality of life (HRQoL), daily life and functioning of survivors^4–6^. An important area for research, therefore, is to identify modifiable lifestyle characteristics such as body composition that could help to ameliorate these health problems experienced by CRC survivors, to improve their HRQoL.

Obesity and overweight, often assessed based on body mass index (BMI), have reached epidemic proportions globally and are accompanied by a range of serious health consequences including cancer^7,8^. One of the cancer prevention recommendations of the World Cancer Research Fund/American Institute for Cancer Research (WCRF/AICR), therefore, is to have a healthy weight and avoid weight gain in adult life based on both BMI and waist circumference^8^. This recommendation also applies to cancer survivors. However, to date, specific evidence with regards to body composition and health outcomes including HRQoL, fatigue and CIPN in cancer survivors including CRC survivors has been inconsistent.

Most studies in long-term (> 5 years) CRC survivors, including one longitudinal study, found that a higher BMI, and in one study also a higher waist circumference^9^, was associated with lower HRQoL and more fatigue^9–17^. Two cross-sectional studies found no association between BMI and physical, mental, and overall quality of life, while one of these studies reported higher cognitive scores and lower levels of fatigue among overweight/obese survivors compared to normal weight survivors^18,19^. Two other studies examined body composition measures based on computed tomography (CT) scans^10,11^. One study found that an increase in CT-based muscle mass during treatment was associated with less fatigue during treatment^11^. In contrast, another study found no association between CT-based visceral obesity and sarcopenia at diagnosis with long-term HRQoL and fatigue^10^. To our knowledge, no studies to date have investigated associations between body composition and CIPN.

Most studies up to date have been cross-sectional and mainly focused on BMI^12–19^. Longitudinal data are needed to evaluate whether post-treatment changes in body composition including both measures of adipose tissue and of muscle mass and muscle function are associated with improvements in HRQoL, fatigue and CIPN. In this longitudinal study of CRC survivors, we therefore aimed to describe whether and how BMI, waist circumference and fat percentage as anthropometric measures of adipose tissue, and mid upper arm muscle circumference (MUAMC) and handgrip strength as measures of muscle mass and muscle function, change over time from diagnosis up to 24 months post-treatment. Moreover, longitudinal associations of these measures were assessed with HRQoL, fatigue, and CIPN from 6 weeks to 24 months post-treatment.

## Methods

### Study design and population

**Figure 1:**
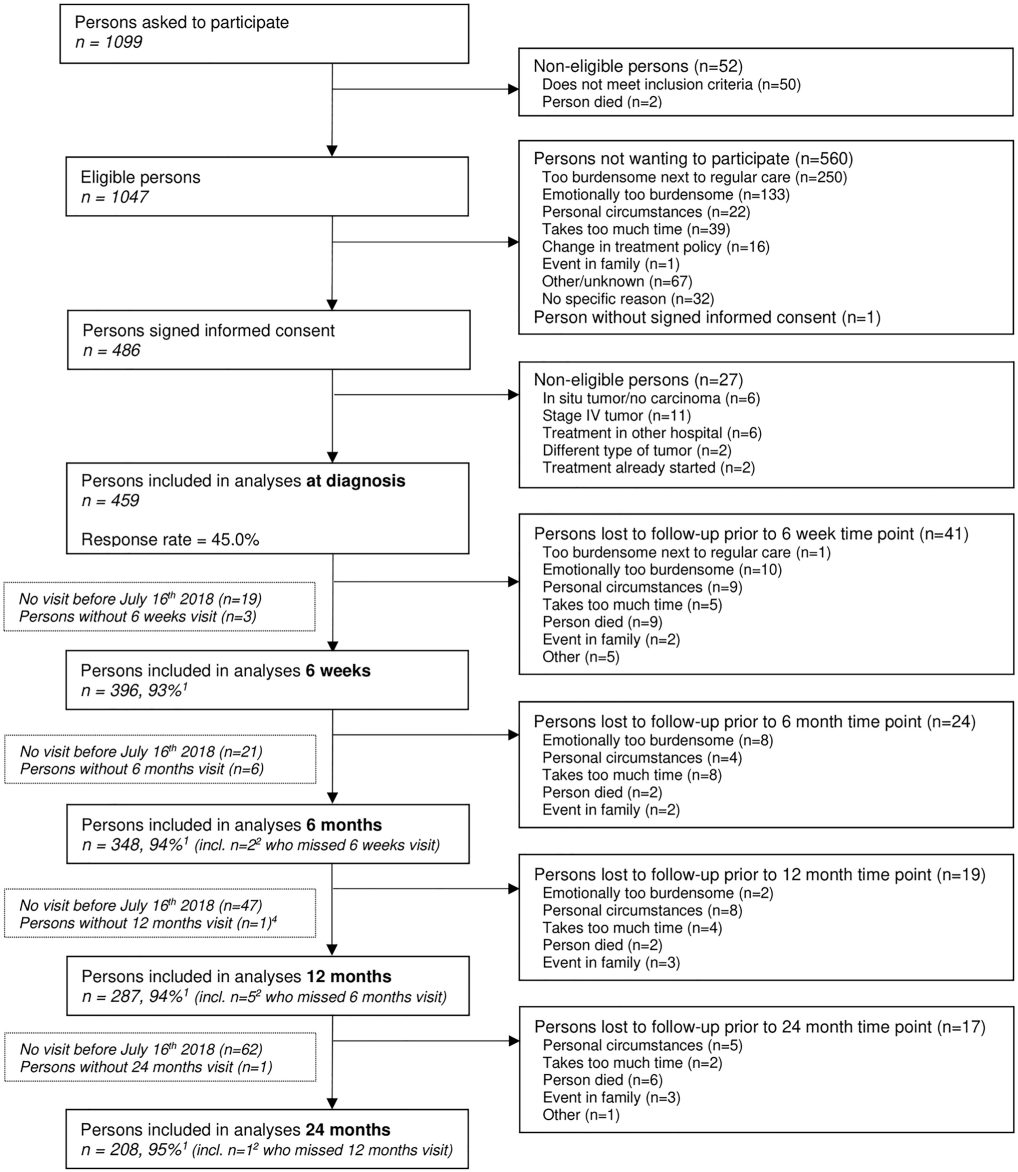
Flow-diagram of inclusion of individuals within the EnCoRe study and included in the analyses presented in this paper. Data of home visits performed before July 16th 2018 were included in the analyses. ^1^Response rate post-treatment = (persons included)/(persons included + persons lost to follow − persons died), ^2^Of the three persons without 6 weeks follow-up visits, one person did not have a 6 months follow-up visit before July 16th 2018. Of the six persons without 6 months follow-up visits, one person did not have a 12 months follow-up visit before July 16th 2018.

Data were used from the Energy for Life after ColoRectal cancer (EnCoRe) study, an ongoing prospective cohort of CRC survivors in the Netherlands (Netherlands Trial Register number: NL6904) that was initiated in 2012^20^. Patients diagnosed with stage I–III CRC at the Maastricht University Medical Center+, VieCuri Medical Center, and Zuyderland Medical Center were recruited. Not eligible for participation were stage IV patients, as well as patients younger than 18 years old, patients not residing in the Netherlands, non-Dutch speaking and reading patients, and patients with comorbidities obstructing successful participation (e.g. Alzheimer’s disease). Trained dietitians collected data during home visits at diagnosis and at 6 weeks, 6 months, 12 months, and 24 months post-treatment. Data collected up until July 2018 were used for the current analysis. The data included information on 459 participants (response rate at diagnosis: 45%) with follow-up measurements at 6 weeks (n = 396), 6 months (n = 348), 12 months (n = 287) and 24 months (n = 208) post-treatment (Fig. 1). Response rates for follow-up visits were all above 91%, and the decrease in number of participants during the follow-up measurements was mainly due to the fact that not all participants had yet reached all post-treatment time points in July 2018.

### Body composition

In accordance with standard operating procedures, trained research dietitians conducted extensive anthropometric measurements during home visits at every time point. Body weight (to the nearest 0.1 kg) was measured in light clothing without shoes on an electronic scale (Seca Ltd, Birmingham, UK type 861). Height (to the nearest 0.1 cm) was measured in duplicate at diagnosis with the subject standing barefoot with heels together, arms at the side, legs straight, shoulders relaxed, and head in the Frankfort horizontal plane with a portable stadiometer. BMI was calculated as weight (kg) divided by squared mean height (m^2^). BMI was categorized according to the WHO criteria as normal weight (18.5 ≤ BMI < 25 kg/m^2^), overweight (25 BMI < 30 kg/m^2^), or obesity (BMI ≥ 30 kg/m^2^)^21^.

As an estimate of visceral adiposity, waist circumference (to the nearest 0.1 cm) was measured in duplicate with a circumeter (type: 05335, Premed) midway between the lower rib margin and the ileac crest^22^, and the mean value was used for further analyses. An increased waist circumference was defined as > 102 cm for men and > 88 cm for women^21^.

Skinfold thickness (to the nearest 0.2 mm) was measured in triplicate at the dominant side of the body using Holtain skinfold calipers^23^ (range 0.00–50.00 mm) at the following sites: triceps; biceps; subscapular; and supra-iliac. The sum of median values of the four skinfolds was used to calculate body fat percentage based on the Durnin–Womersley calculations with Siri equation^24^.

Mid upper arm circumference (MUAC) (to the nearest 0.1 cm) of the dominant arm was measured in duplicate with a circumeter at a point midway between the acromion process and the olecranon process^25^. MUAMC was calculated based on the mean MUAC and the median triceps skinfold thickness using the standard formula: *MUAMC* = *MUAC* − (3.1415 × *triceps* *skinfold* *thickness*)^26^.

Handgrip strength was assessed as a proxy of overall muscle strength and function^27,28^. Measurement of maximum grip strength (to the nearest kg) was performed with the dominant hand using a handheld dynamometer, with the participant in the seated position and the elbow flexed at 90°. The participant was instructed to squeeze the handle as hard as possible for 3–5 s. The measurement was repeated after a brief recovery period, and the highest value was used for further analyses.

### HRQoL, fatigue and CIPN

The widely used and well validated European Organization for the Research and Treatment of Cancer Quality of Life Questionnaire-Core 30 (EORTC QLQ-C30) was used to measure cancer-specific HRQoL^29^. The EORTC QLQ-C30 is a 30-item questionnaire comprised of five functioning scales (physical, role, cognitive, emotional, and social functioning), three symptom scales (fatigue, pain, and nausea and vomiting), a global health/QoL scale, and a number of items for specific cancer-/treatment-related symptoms. In addition, a summary score (SumSc) can be calculated from the mean of 13 of the 15 subscale scores (excluding the financial difficulties and global QoL questions)^30^. All scores were linearly transformed to a 0–100 scale, with higher scores on the functioning scales, global QoL and SumSc reflecting better functioning or HRQoL, whereas higher symptom scale scores indicate more symptoms (i.e. worse fatigue). HRQoL outcomes included in the current analyses were global QoL, physical functioning, role functioning, social functioning, and the summary score.

Next to the EORTC QLQ-C30 fatigue subscale, fatigue was more comprehensively assessed by the Checklist Individual Strength (CIS). The CIS was originally developed and validated in patients with chronic fatigue syndrome^31^, but has also been applied in cancer survivors^32^. This 20-item questionnaire with a 7-point Likert scale includes four subscales reflecting different aspects of fatigue: subjective feelings of fatigue (8 items, score range 8–56), concentration problems (5 items, range 5–35), reduced motivation (4 items, score range 4–28), and reduced physical activity (3 items, score range 3–21). In addition, a total score was derived by the summation of the items, ranging from 20 to 140 points. Higher scores indicate worse fatigue on all scales. Fatigue outcomes included the EORTC fatigue subscale and the CIS total score and subscales reduced physical activity and subjective fatigue.

The EORTC QLQ-CIPN20 was used to measure complaints related to CIPN. This questionnaire consists of sensory (9 items), motor (7 items) and autonomic subscales (2 items), and a summary score (18 items)^33^. Scores for separate scales were linearly converted to a 0–100 scale^34^. Higher scores on the CIPN20 scales indicate more CIPN-related complaints. The summary score and all subscales were used for the current analyses.

### Lifestyle, clinical, and sociodemographic factors

Clinical information (i.e. cancer stage, surgery/chemotherapy/radiotherapy treatment, and tumour site), age and sex were retrieved from medical records. Self-reported data were collected on highest attained education level (only at diagnosis), and current smoking status and presence of comorbidities at all time points. The presence of comorbidities at post-treatment time points was collected using the Self-Administered Comorbidity Questionnaire^35^. The Short QUestionnaire to ASsess Health-enhancing physical activity (SQUASH) was used to determine hours/week of moderate-to-vigorous physical activity (MVPA) at every time point^36,37^. Further, prolonged sedentary behaviour (hours/day), i.e. time accrued in sedentary bouts with a duration of at least 30 min, was objectively assessed using the validated tri-axial MOX activity monitor, worn by the participants for 7 consecutive days at each post-treatment time point (Maastricht Instruments B.V., NL)^38^. Dietary intake was measured through 7-day food diaries collected at each post-treatment time point^39^. A diet quality score was calculated based on the five nutrition recommendations of the WCRF/AICR guidelines (continuous score 0–5), including intake of fruits and vegetables, fiber, red and processed meat, ultra-processed food, sugar-sweetened drinks and alcoholic drinks^8,40^. The Hospital Anxiety and Depression Scale (HADS) was used to determine depression and anxiety-related complaints at every post-treatment time point^41^.

### Statistical analyses

Descriptive statistics including means and standard deviations (SDs) for normally distributed variables, medians and interquartile ranges for non-normally distributed variables, or frequencies and percentages for categorical variables were calculated to describe main sample characteristics including body composition measures and patient-reported outcomes, overall and by sex.

Longitudinal analyses including modelling of the development of exposures and outcomes over time and modelling of associations between body composition and HRQoL, fatigue and CIPN were assessed using linear mixed models. A random intercept for each subject was added to all models. The use of random slopes was tested with a likelihood-ratio test; when the model improved significantly random slopes were added.

For describing longitudinal trajectories (modelling over time) for anthropometric measures and CIPN, time was modelled as a categorical variable, represented by four dummy variables because of a non-linear relationship over time between diagnosis and 24 months post-treatment. The regression coefficients of the dummy variables indicate the change in anthropometric measures and CIPN between the time points and the reference category (at 6 weeks). Since HRQoL and fatigue were not measured at diagnosis and were hypothesized to follow a linear relationship over time, these outcomes were modelled as dependent variables in linear mixed models including time as a continuous covariate per 6 months from 6 weeks up to 24 months post-treatment. The regression coefficient of the time covariate thus indicates the mean change per 6 months in HRQoL and fatigue in the period from 6 weeks up to 24 months after treatment.

Longitudinal associations between each body composition measure and HRQoL, fatigue and CIPN outcomes between 6 weeks and 24 months post-treatment were adjusted for a priori defined confounders that included fixed (time-invariant) confounders comprising age at enrolment (years), sex (male/female), education level (low, medium, high), chemotherapy (yes, no), radiotherapy (yes, no), and the body composition measure of interest at diagnosis, as well as time-variant confounders measured at all post-treatment time points comprising MVPA (hours/week), prolonged sedentary behaviour (hours/day), number of comorbidities (0, 1, ≥ 2), smoking (current, former and never), time since diagnosis (months), psychological distress (HADS score), and dietary quality score (0–5). The inclusion of post-treatment BMI as a potential confounder in models with MUAMC and handgrip strength as outcomes resulted in changes < 10% in regression coefficients, and therefore post-treatment BMI was not included as covariate in these models. Inter- and intra-individual associations were disaggregated by adding centred person-mean values to the model to estimate inter-individual associations (i.e. due to differences in body composition measures between individuals), and individual deviations at each time point from the person-mean value to estimate intra-individual associations (i.e. due to changes in body composition measures within individuals)^42^. The body composition measures were modelled as continuous variables per 0.5 SD, which can be considered a clinically relevant contrast^43^. CIPN outcomes were only analysed for the subgroup of patients who received chemotherapy^44^.

Potential interaction between body composition measures and sex, chemotherapy, and comorbidity was explored by including interaction terms in linear mixed models. Sex-stratified, chemotherapy-stratified and comorbidity-stratified analyses were performed when interaction terms were statistically significant. BMI-stratified analyses (≤ 25 kg/m^2^ and > 25 kg/m^2^) were performed to explore whether associations were independent of BMI at diagnosis.

Statistical analyses were performed using Stata 15.0 (StataCorp. 2017. College Station, TX) with statistical significance set at p < 0.05 (two-sided).

### Ethics approval

The EnCoRe study has been approved by the Medical Ethics Committee of the Academic Hospital Maastricht and Maastricht University, The Netherlands (METC 11-3-075). The study was performed in accordance with the Declaration of Helsinki.

### Informed consent

All participants gave written informed consent.

## Results

### Participant characteristics

The characteristics of the total population (n = 459) at diagnosis, and the characteristics of the follow-up time points are presented in Table 1. At diagnosis, participants were on average 67 years old (SD: 9.1) and two-thirds were men (n = 303, 66%). A total of 290 participants were diagnosed with colon cancer (63%) and 169 with rectal cancer (37%). In addition, 31% of the participants had stage I, 24% had stage II, and 46% had stage III CRC. Over one-third (40%) of the participants received chemotherapy and 25% received radiotherapy. Characteristics of the sample remained similar across all post-treatment time points except for education, where the proportion of participants reporting having had a low education changed from 29% at diagnosis to 21% at 24 months post-treatment.

**Table 1:** Demographic, lifestyle, and clinical characteristics of colorectal cancer survivors at diagnosis, and 6 weeks, 6 months, 12 months, and 24 months post-treatment.

	Diagnosis (n = 459)	6 weeks post-treatment (n = 396)	6 months post-treatment (n = 348)	12 months post-treatment (n = 287)	24 months post-treatment (n = 208)
**Sex** (male) [n (%)]	303 (66.0)	270 (68.2)	236 (67.8)	196 (68.3)	142 (68.3)
**Age** (years) [mean (SD)]	66.9 (9.1)	67.0 (9.1)	67.2 (9.23)	67.4 (9.2)	68.1 (9.2)
**Body mass index, kg/m**^**2**^** [mean (SD)]**	28.3 (4.7)	27.8 (4.6)	28.3 (4.7)	28.7 (4.8)	28.3 (4.6)
Underweight: < 18.5	2 (0.4)	2 (0.5)	0 (0.0)	1 (0.4)	1 (0.5)
Healthy weight: 18.5–24.9	111 (24.3)	117 (29.6)	90 (25.9)	62 (21.9)	49 (24.0)
Overweight: 25–29.9	201 (44.0)	173 (43.8)	151 (43.5)	130 (45.9)	85 (41.7)
Obese: ≥ 30	143 (31.3)	103 (26.1)	106 (30.6)	90 (31.8)	69 (33.8)
**Smoking [n (%)]**					
Never	139 (31.0)	118 (30.5)	98 (28.7)	76 (27.6)	57 (29.1)
Former	255 (56.8)	235 (60.7)	213 (62.5)	172 (62.6)	120 (61.2)
Current	55 (12.3)	34 (8.8)	30 (8.8)	27 (9.8)	19 (9.7)
**Education [n (%)]**					
Low	130 (29.0)	107 (27.1)	91 (26.2)	73 (25.5)	45 (21.6)
Medium	168 (37.4)	149 (37.7)	137 (39.5)	114 (39.9)	89 (42.8)
High	151 (33.6)	139 (35.2)	119 (34.3)	99 (34.6)	74 (34.6)
**Cancer type [n (%)]**					
Colon	290 (63.2)	250 (63.1)	222 (63.8)	181 (60.6)	126 (60.6)
Rectosigmoid and rectum	169 (36.8)	146 (36.9)	126 (36.2)	106 (36.9)	82 (39.4)
**Tumour stage [n (%)]**					
Stage I	141 (30.7)	124 (31.3)	109 (31.3)	97 (33.8)	71 (34.1)
Stage II	108 (23.5)	100 (25.3)	86 (24.7)	69 (24.0)	52 (25.0)
Stage III	210 (45.8)	172 (43.4)	153 (44.0)	121 (42.2)	85 (40.9)
**Treatment [n (%)]**					
Surgery (yes)	412 (89.8)	354 (89.4)	317 (91.1)	259 (90.2)	186 (89.4)
Chemotherapy (yes)	184 (40.1)	155 (39.1)	134 (38.5)	107 (37.3)	79 (38.0)
Radiotherapy (yes)	116 (25.3)	101 (25.5)	88 (25.3)	73 (25.4)	55 (26.4)

*BMI* body mass index, *SD* standard deviation.

^a^Percentages may not add to 100 due to rounding off.

^b^Data missing for two participants (female).

### Changes in body composition measures between diagnosis and 24 months post-treatment

**Figure 2:**
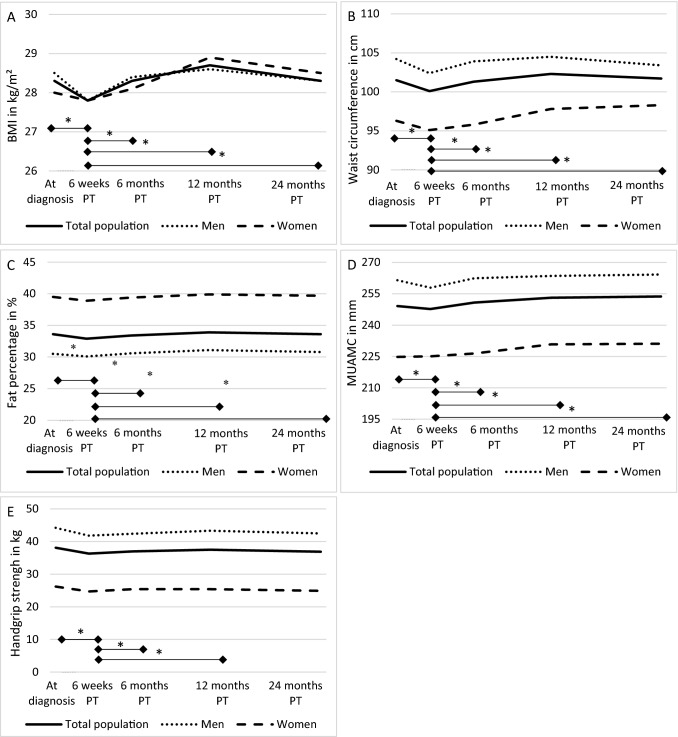
Course of BMI (**A**), waist circumference (**B**), fat percentage (**C**), mid upper arm muscle circumference (**D**), and handgrip strength (**E**) from diagnosis up to 24 months post-treatment (PT) in colorectal cancer survivors in the EnCoRe study. Treatment took place between diagnosis and 6 weeks post-treatment. *Indicates statistically significant differences (p < 0/05) between time points indicated by the horizontal line. *BMI* body mass index, *MUAMC* mid upper arm muscle circumference, *PT* post-treatment.

At diagnosis, mean BMI was 28.3 kg/m^2^ (SD: 4.7 kg/m^2^) and 24.7% of participants were classified as normal weight, 43.0% as overweight, and 31.3% as obese. At 6 weeks post-treatment, a decreased mean BMI of 27.8 kg/m^2^ (SD: 4.6 kg/m^2^) was observed, and 30.1% were classified as normal weight, 43.8% as overweight, and 26.1% as obese. After 6 weeks post-treatment, BMI increased to an average of 28.3 kg/m^2^ (SD: 4.6 kg/m^2^) at 24 months post-treatment (Fig. 2a). All body composition measures showed a similar trend over time, first decreasing between diagnosis and 6 weeks post-treatment and thereafter slowly increasing up to 24 months after treatment (Fig. 2b–e). When using indicator variables for different time points, BMI was found to be significantly higher at diagnosis (β: 0.6 kg/m^2^; 95% CI 0.5, 0.7) and at 6 months (0.6; 0.4, 0.7), 12 months (0.9; 0.8, 1.1), and 24 months post-treatment (0.9; 0.8, 1.1), compared to 6 weeks post-treatment (Fig. 2a). Similar statistically significant results were obtained for waist circumference (Fig. 2b), body fat percentage (Fig. 2c) and MUAMC (Fig. 2d). For handgrip strength (Fig. 2e) the highest value was measured at diagnosis relative to 6 weeks post-treatment as reference (β: 2.3 kg; 95% CI 1.9, 2.8). Afterwards, handgrip strength slightly increased from 6 weeks to 6 months (0.7; 0.3, 1.2) and 12 months (1.0; 0.5, 1.5), but was similar at 24 months (− 0.1; − 0.6, 0.5). Figure 2 also shows that women generally had a similar BMI, higher body fat percentage and lower handgrip strength, MUAMC, and waist circumference in comparison to men at all time points.

### Changes in quality of life and fatigue between 6 weeks and 24 months post-treatment

Mean scores of global quality of life (β per 6 months: 0.9; 95% CI 0.3, 1.5), physical functioning (1.7; 1.2, 2.2), role functioning (3.3; 2.4, 4.2), and social functioning (2.1; 1.4, 2.7) increased statistically significantly from the 6 weeks to the 24 months post-treatment time point.

The total fatigue score followed a significant decline between 6 weeks and 24 months post-treatment (β per 6 months: − 2.2; 95% CI − 2.9, − 1.4), and this was also observed for the subscales subjective fatigue (− 1.1; − 1.5, − 0.8) and reduced activity (− 0.6; − 0.8, − 0.4). Fatigue measured by the EORTC also significantly decreased from 6 weeks to the 24 months post-treatment time point (− 2.2; − 2.9, − 1.6).

### Changes in CIPN outcomes between diagnosis and 24 months post-treatment

**Figure 3:**
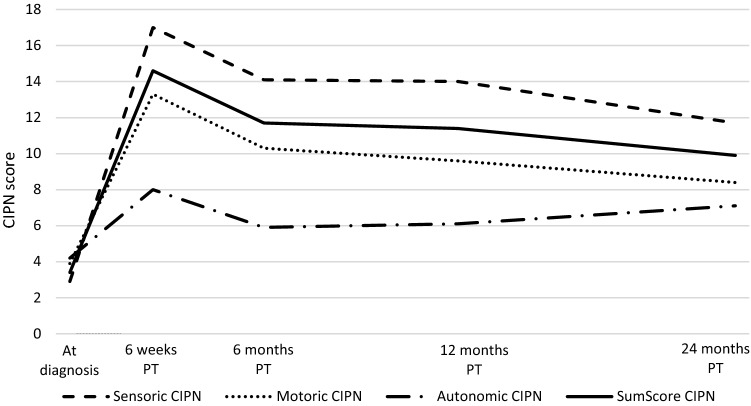
Course of CIPN from diagnosis up to 24 months post-treatment (PT) in colorectal cancer survivors having received chemotherapy in the EnCoRe study. Treatment took place between diagnosis and 6 weeks post-treatment. Course is shown for the different subscales used in the EORTC-CIPN20. *CIPN* chemotherapy-induced peripheral neuropathy, *PT* post-treatment.

Among CRC survivors who received chemotherapy, CIPN summary and subscale scores changed over time from diagnosis up to 24 months post-treatment (Fig. 3). Highest mean scores were observed at 6 weeks post-treatment, followed by a steep decrease to 6 months post-treatment and thereafter a more gradual decrease up to 24 months post-treatment. When using indicator variables for different time points with 6 weeks post-treatment as reference, CIPN scores were significantly lower at 24 months for the summary score (β: − 5.0; 95% CI − 7.3, − 2.6), motor subscale (− 4.8; − 7.4, − 2.3) and sensory subscale (− 6.0; − 9.1, − 2.9). Nevertheless, mean CIPN scores were significantly higher at 24 months after treatment in comparison to CIPN scores at diagnosis (Fig. 3) and were also higher compared to the normative population^45^, indicating that many CRC survivors are still suffering from CIPN complaints at 24 months after treatment.

### Longitudinal associations of anthropometric measures with HRQoL, fatigue and CIPN

**Figure 4:**
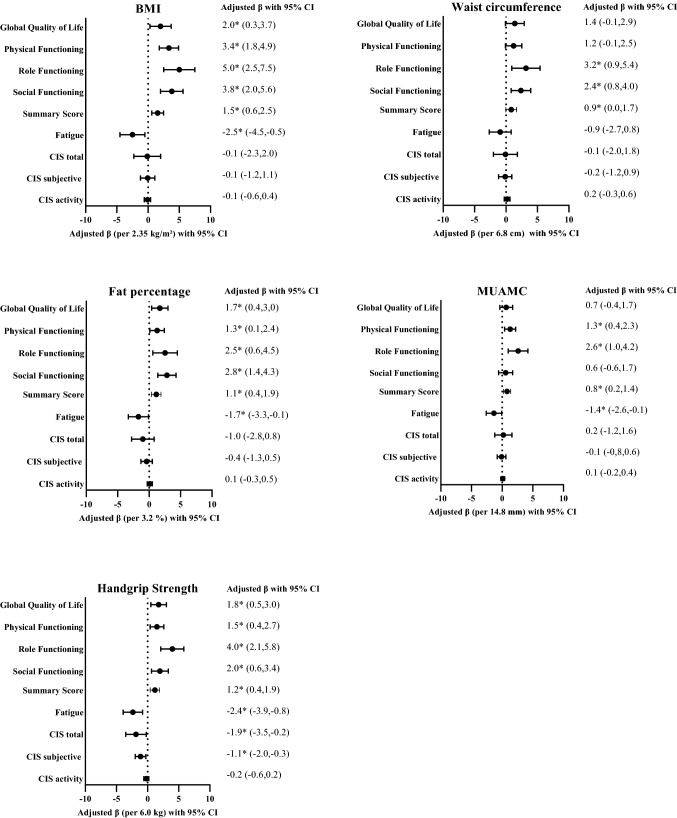
Forest plots showing the confounder-adjusted betas (β) and 95% confidence intervals (CI) for the overall longitudinal associations of the five body composition measures (BMI, waist circumference, fat percentage, MUAMC, and handgrip strength) in relation to health-related quality of life and fatigue outcomes in stage I–III colorectal cancer survivors followed-up from 6 weeks to 2 years after treatment. *Indicates statistically significant associations (p < 0.05) between body composition measure and health-related quality of life and fatigue outcomes.

In confounder-adjusted models assessing overall longitudinal associations from 6 weeks to 24 months after CRC treatment (Fig. 4 and Supplement Table 1), a 0.5 SD higher BMI, waist circumference, and fat percentage was significantly associated with several HRQoL outcomes. For example, BMI was associated with better global QoL (β per 2.35 kg/m^2^ 1.2; 95% CI 0.3, 4.1), physical functioning (3.3; 1.8, 4.9), role functioning (5.0; 2.5, 7.5), and social functioning (3.8; 2.0, 5.6). A higher BMI was also associated with less EORTC-fatigue (− 2.5; − 4.5, − 0.5). No significant associations were found for fatigue measured by the CIS.

A 0.5 SD higher handgrip strength was associated with better global QoL (β per 6 kg: 1.8; 95% CI 0.5, 3.0), better physical functioning (1.5; 0.4, 2.7), role functioning (4.0; 2.1, 5.8), and social functioning (2.0; 0.6, 3.4), and with less fatigue (− 2.4; − 3.9, − 0.8). Similar directions of associations were observed for MUAMC, although most were non-statistically significant (Fig. 4 and Supplement Table 1). With regards to fatigue measured by the CIS, a higher handgrip strength was longitudinally associated with lower total fatigue (β per 6 kg: − 1.9; 95% CI − 3.5, − 0.2) and subjective fatigue (− 1.1; − 2.0, − 0.3).

Separate models testing inter- and intra-individual associations (Supplement Table 1) showed that the associations for BMI were mostly driven by the intra-individual component, indicating that an increase in BMI over time within individuals and not a difference in BMI between individuals was predominantly associated with better HRQoL and less fatigue over time. In particular, intra-individual analyses showed that an increase in BMI was significantly associated with better global QoL (β per 2.35 kg/m^2^: 2.1; 95% CI 0.0, 4.1), physical functioning (3.0; 1.4, 4.7), role functioning (6.5; 3.2, 9.9), and social functioning (4.7; 2.3, 7.0), and less fatigue (− 3.3; − 5.8, − 0.8). Most inter-individual associations had the same direction as intra-individual associations, although the regression coefficients were smaller and mostly non-significant. The same pattern was seen for the other adipose and muscle mass measures (Supplement Table 1).

Significant associations with CIPN in participants who received chemotherapy were found for handgrip strength only (Supplement Table 2). In particular, a higher handgrip strength was associated with a decrease in symptoms on the summary CIPN score (β per 6 kg: − 3.3; 95% CI − 4.9, − 1.7), motor subscale (− 3.8; − 5.5, − 2.1), and sensory subscale (− 3.5; − 5.6, − 1.4). These longitudinal associations appeared mostly driven by inter-individual associations.

### Additional interaction analyses

Statistically significant interactions between handgrip strength and chemotherapy were found for physical functioning (p = 0.006) and role functioning (p = 0.006). When stratifying the analyses by chemotherapy (yes vs. no), higher handgrip strength was associated with better physical functioning (β per 6 kg: 2.1; 0.7, 3.5) in the non-chemotherapy group, whereas this association was not found in the chemotherapy group (0.4; − 1.4, 2.2). There was also a difference in the non-chemotherapy group (6.1; 3.6, 8.5) versus chemotherapy group (1.6; − 1.4, 4.5) for role functioning.

In addition, a significant interaction between handgrip strength and comorbidities was found for physical functioning (p = 0.022) and role functioning (p = 0.003). Stratified analyses for comorbidity showed that higher handgrip strength was associated with better physical functioning in participants with ≥ 2 comorbidities (β per 6 kg: 2.7; 1.0, 4.4) but not in participants with 0–1 comorbidities (0.7; − 0.8, 2.1). There was also a difference in participants with ≥ 2 comorbidities (6.6; 3.7, 9.4) versus participants with 0–1 comorbidities (2.3; − 0.1, 4.7) for role functioning.

No significant statistical interactions were observed for sex with any of the body composition measures. Lastly, the BMI-stratified analyses showed similar associations across both groups (≤ 25 kg/m^2^ and > 25 kg/m^2^).

## Discussion

Within this longitudinal study of stage I–III CRC survivors, we described changes over time in body composition measures reflecting adipose tissue (BMI, waist circumference, fat percentage) and muscle mass and muscle function (MUAMC, handgrip strength) from diagnosis up to 24 months after treatment. All body composition measures showed a similar trend over time, first decreasing between diagnosis and 6 weeks post-treatment and thereafter slowly increasing up to 24 months after treatment. In confounder-adjusted analyses, we observed that increases in body composition measures of adipose tissue and muscle function were longitudinally associated with increases in HRQoL outcomes and decreases in fatigue from 6 weeks up to 24 months post-treatment. These associations appeared to be mainly driven by within-person changes over time, indicating that, e.g., post-treatment increases in BMI over time within individuals, instead of a difference in BMI between individuals, were associated with better HRQoL and less fatigue over time. Additionally, increased handgrip strength was longitudinally associated with decreased CIPN complaints in chemotherapy-treated participants, which could mainly be attributed to between-person differences in handgrip strength over time.

To the best of our knowledge, this is the first study that assessed longitudinal relationships between a comprehensive set of body composition measures and HRQoL, fatigue and CIPN in CRC survivors, from 6 weeks up to 24 months post-treatment. Several cross-sectional studies previously reported increased BMI to be related with decreased HRQoL, which is in contrast with our findings. These cross-sectional studies were mostly conducted among long-term CRC survivors (> 5 years)^9,12,13,16,17^, whilst we examined the first 2 years after treatment in a longitudinal study. In accordance with our study, one cross-sectional study in CRC survivors who were on average 2 years post-diagnosis found a higher BMI to be associated with lower fatigue and increased cognitive functioning^18^. Another study observed an association between higher skeletal muscle index and less fatigue during treatment^11^. Altogether, inconsistencies in findings between previous studies as well as with our findings in the present study indicate that timing of the association, as well as employed measures of body composition and patient-reported outcomes, and the design of the study are important factors that need to be taken into account when interpreting the relation between body composition and patient-reported outcomes after CRC. On the long-term, overweight and obesity might be related to worse HRQoL, observed by most long-term CRC survivors studies^9,12,13,16,17^ and also observed in the general population^15,46^. However, this study highlights that within the first years after diagnosis and treatment, recovery of treatment-induced detriments of body composition influencing both adipose and muscle mass, as well as muscle function, may be associated with improvements in HRQoL and decreases in fatigue. Importantly, all analyses were adjusted for time since treatment, therefore the associations we observed cannot be explained by both body composition and HRQoL increasing over time. Altogether, we consistently observed that increasing estimates for parameters reflecting muscle mass and muscle function and, somewhat unexpectedly, also parameters reflecting adipose tissue were favourably related to quality of life, functioning, and fatigue. The changes observed in body composition over time were the same for normal weight people as for overweight and obese people (data not shown), indicating that recovery of body composition after the immediate cancer treatment period characterizes all CRC survivors regardless of pre-treatment body composition. Interestingly, the longitudinal relations between body composition and HRQoL observed in our study appeared mostly driven by intra-individual associations, further emphasizing the importance of recovery of all aspects of body composition for improvements of patient-reported outcomes in the first couple of years after treatment.

Although we observed a number of statistically significant longitudinal associations, the associations appeared to be rather small when considering previously published guidelines for interpreting effect sizes for patient-reported outcomes^47–49^. Nevertheless, the consistent significant longitudinal associations across all body composition and HRQoL outcomes using various body composition measures underline the importance of our findings. These findings could have important implications for the WCRF/AICR lifestyle recommendations for cancer survivors regarding a healthy weight and HRQoL, for instance by also emphasizing the relevance of recovery from negative effects of the cancer and treatment on body composition during the first 2 years after CRC treatment when focusing on HRQoL. At the moment, the recommendations for cancer survivors focus on having a healthy body weight for the prevention of cancer and this could imply using the weight loss after treatment as a momentum to obtain or come closer to a healthy weight. Although a healthy weight is important for recurrence and survival^50^, our results indicate that within the first 2 years after treatment, recovery of body composition including both adipose and muscle mass and muscle function may be beneficial to recover quality of life and functioning and decrease fatigue and CIPN symptoms in CRC survivors.

A major strength of this study was that information on body composition was not obtained through self-report but collected by trained dietitians who performed anthropometric measurements and using the identical measurement instrument (i.e. the same weighing scale as the previous measurement), increasing the validity relative to self-reported measurements where there is a tendency for height to be overestimated and weight to be underestimated^51^. Moreover, the availability of extensive measures of adipose tissue, muscle mass and muscle function are of added value. Other strengths of our study included the high response rates during follow-up (> 90%), the limited number of missing data resulting from intensive data collection methods, and availability of extensive data on potential confounders and effect modifiers. Although numbers decreased over time because participants had not yet reached all time points, available data from all participants were included in the mixed models since this analysis technique efficiently deals with random missings. Furthermore, the mixed models enabled disentangling of inter- and intra-individual associations, thereby providing additional insights into the nature of the longitudinal associations.

There are also limitations that should be considered. Based on these observational data, we cannot be sure of the direction of associations between body composition and HRQoL, fatigue and CIPN. Intervention studies will be necessary to infer causality, e.g. whether for example pre- or post-treatment strength training can prevent or decrease CIPN complaints in chemotherapy-treated CRC patients. In addition, the limited response rate at diagnosis (45%) might have resulted in a selection bias. Participants with worse body composition and lower quality of life were possibly less likely to participate and this may have led to an attenuation of associations. We also observed that participants with a high education appeared to be slightly more likely to stay in the study compared to participants with a low education (Table 1). This potential for selective loss to follow-up might have also led to an attenuation of associations. Moreover, because we had no information on HRQoL and fatigue at diagnosis as well as complete follow-up for recurrences during post-treatment follow-up, we were not able to adjust for these potential confounders. Finally, we cannot rule out the possibility of false positives due to the large number of tests performed.

In conclusion, our results suggest that in the first 24 months after CRC treatment, increases in adipose tissue and muscle mass and muscle function, potentially indicating post-treatment recovery of body composition, are associated with improved HRQoL, less fatigue and CIPN, independent of BMI status at diagnosis. Therefore, in clinical practice, the focus should also be on recovery from the impact of treatment and on restoring body tissues and function especially with regards to improving HRQoL and decreasing fatigue and CIPN complaints. Future studies are needed to further investigate the duration of this potential recovery period and the implications post-treatment changes in adiposity and muscle mass and function can have on other risks such as recurrence and survival. This can ultimately contribute to more specific guidelines for CRC survivors in order to improve their health and well-being in the years after treatment.

## Supplementary Information

Supplementary Table 1.

Supplementary Table 2.

## Data Availability

Data described in the manuscript, code book, and analytic code will be made available upon request pending (e.g., application and approval, payment, other). Requests for data of the EnCoRe study can be sent to Dr. Martijn Bours, Department of Epidemiology, GROW-School for Oncology and Developmental Biology, Maastricht University, the Netherlands (email: m.bours@maastrichtuniversity.nl).

## Abbreviations

AICR: American Institute for Cancer Research
BMI: Body mass index
CIPN: Chemotherapy-induced peripheral neuropathy
CIS: Checklist individual strength
CRC: Colorectal cancer
CT: Computed tomography
EnCoRe: Energy for Life after ColoRectal cancer
EORTC QLQ-C30: European Organization for the Research and Treatment of Cancer Quality of Life Questionnaire-Core 30
HRQoL: Health-related quality of life
MUAMC: Mid upper arm muscle circumference
MUAC: Mid upper arm circumference
MVPA: Moderate-to-vigorous physical activity
SDs: Standard deviations
SQUASH: Short QUestionnaire to ASsess Health-enhancing physical activity
WCRF: World Cancer Research Fund
