# Supplementary material for: Increases in adipose tissue and muscle function are longitudinally associated with better quality of life in colorectal cancer survivors
Source: Sci Rep. 2021 Jun 14;11:12440. doi: 10.1038/s41598-021-91709-y (PMC8203762; doi:10.1038/s41598-021-91709-y)
Supplement: Supplementary file 1 — Supplementary Table 1. [file 41598_2021_91709_MOESM1_ESM.docx]

Supplement Table 1 Linear mixed models of the five body composition measures (BMI, waist circumference, fat percentage, MUAMC and handgrip strength) in relation to health-related quality of life and fatigue

|  |  | EORTC QLQ-C30 | | | | | | Checklist individual strength | | |
| --- | --- | --- | --- | --- | --- | --- | --- | --- | --- | --- |
|  | Global QoL  (0-100) | Physical functioning  (0-100) | Role functioning  (0-100) | Social functioning  (0-100) | Summary score  (0-100) | Fatigue (EORTC)  (0-100) | Fatigue (CIS)  (20-140) | Subjective fatigue (CIS)  (8-56) | Activity fatigue (CIS)  (3-21) |  |
|  | β (95% CI) | β (95% CI) | β (95% CI) | β (95% CI) | β (95% CI) | β (95% CI) | β (95% CI) | β (95% CI) | β (95% CI) |  |
| BMI  (per 2.35 kg/m²)^b^ | Unadjusted | -0.53  (-1.26,0.20) | -0.28  (-1.04,0.48) | -0.39  (-1.35,0.57) | 0.27  (-0.49,1.04) | -0.06  (-0.55,0.44) | 0.17  (-0.72,1.07) | 0.70  (-0.37,1.77) | 0.51*  (0.00,1.03) | 0.15  (-0.05,0.36) |
|  | Adjusted^ab^ | 1.97* (0.28,3.66) | 3.35*  (1.84,4.85) | 5.03*  (2.53,7.53) | 3.84*  (2.04,5.64) | 1.52*  (0.56,2.48) | -2.49*  (-4.52,-0.46) | -0.14  (-2.31,2.04) | -0.06  (-1.24,1.12) | -0.07  (-0.56,0.42) |
|  | Intra^c^ | 2.07* (0.02,4.12) | 3.04*  (1.41,4.66) | 6.53*  (3.17,9.88) | 4.66*  (2.30,7.03) | 1.87*  (0.76,2.99) | -3.30*  (-5.79,-0.81) | 0.04  (-2.52,2.61) | 0.15  (-0.40,0.71) | -0.10  (-0.69,0.49) |
|  | Inter^d^ | 1.69 (-0.92,4.31) | 2.99*  (0.10,5.89) | 3.31  (-0.29,6.91) | 2.80*  (0.15,5.45) | 0.61  (-1.15,2.37) | -1.01  (-4.33,2.31) | -0.57  (-4.46,3.32) | -0.36  (-0.95,1.67) | -0.02  (-0.85,0.82) |
| Waist circumferen-ce (per 6.8 cm)^b^ | Unadjusted | -0.77* (-1.49,-0.05) | -0.39  (-1.14,0.35) | -0.34  (-1.30,0.63) | 0.03  (-0.73,0.80) | -0.09  (-0.58,0.40) | 0.42  (-0.47,1.30) | 0.78  (-0.28,1.83) | 0.46  (-0.05,0.97) | 0.26  (0.05,0.46) |
|  | Adjusted^ab^ | 1.39 (-0.07,2.86) | 1.20  (-0.06,2.47) | 3.17*  (0.94,5.41) | 2.39*  (0.84,3.95) | 0.85*  (0.01,1.69) | -0.93  (-2.71,0.84) | -0.12  (-2.02,1.79) | -0.15  (-1.16,0.86) | 0.16  (-0.27,0.58) |
|  | Intra^c^ | 0.94 (-0.88,2.76) | 1.32  (-0.13,2.77) | 2.68  (-0.34,5.69) | 2.08  (-0.02,4.17) | 0.72  (-0.28,1.72) | -0.69  (-2.93,1.55) | 0.14  (-2.14,2.43) | 0.22  (-0.94,1.39) | 0.18  (-0.34,0.70) |
|  | Inter^d^ | 1.87 (-0.31,4.06) | 0.86  (-1.59,3.31) | 3.41*  (0.39,6.43) | 2.75*  (0.52,4.98) | 1.12  (-0.36,2.60) | -1.32  (-4.11,1.47) | -0.67  (-3.96,2.62) | -0.65  (-2.31,1.01) | 0.11  (-0.60,0.82) |
| Fat percentage (per 3.2 %)^b^ | Unadjusted | 0.11 (-0.60,0.83) | -0.55  (-1.27,0.17) | -0.29  (-1.23,0.66) | 0.04  (-0.70,0.79) | -0.09  (-0.56,0.38) | 0.49  (-0.38,1.36) | 0.35  (-0.68,1.39) | 0.37  (-0.13,0.87) | 0.06  (-0.15,0.26) |
|  | Adjusted^ab^ | 1.68* (0.39,2.96) | 1.26*  (0.12,2.40) | 2.52*  (0.56,4.49) | 2.84*  (1.44,4.25) | 1.13*  (0.38,1.88) | -1.68*  (-3.27,-0.09) | -1.01  (-2.76,0.75) | -0.40  (-1.30,0.49) | 0.11  (-0.27,0.49) |
|  | Intra^c^ | 1.93* (0.31,3.54) | 1.48*  (0.20,2.77) | 3.04*  (0.38,5.71) | 3.37*  (1.50,5.24) | 1.11*  (0.22,1.99) | -1.80  (-3.78,0.17) | -0.62  (-2.64,1.41) | -0.17  (-1.20,0.87) | 0.21  (-0.25,0.68) |
|  | Inter^d^ | 1.26 (-0.81,3.33) | 0.51  (-1.82,2.85) | 1.93  (-0.92,4.78) | 2.19*  (0.11,4.27) | 1.20  (-0.19,2.59) | -1.47  (-4.09,1.14) | -1.50  (-4.60,1.60) | -0.75  (-2.31,0.82) | -0.10  (-0.77,0.56) |

| MUAMC  (per 14.8 mm)^b^ | Unadjusted | 0.16 (-0.52,0.84) | 1.47*  (0.81,2.12) | 0.78  (-0.15,1.71) | 0.35  (-0.38,1.07) | 0.54*  (0.10,0.98) | -1.02*  (-1.85,-0.19) | -0.35  (-1.32,0.61) | -0.20  (-0.67,0.27) | 0.02  (-0.18,0.21) |
| --- | --- | --- | --- | --- | --- | --- | --- | --- | --- | --- |
|  | Adjusted^ab^ | 0.66 (-0.38,1.70) | 1.33*  (0.41,2.25) | 2.57*  (0.99,4.15) | 0.58  (-0.56,1.73) | 0.77*  (0.16,1.37) | -1.35*  (-2.63,-0.06) | 0.22  (-1.19,1.64) | -0.07  (-0.78,0.64) | 0.10  (-0.21,0.42) |
|  | Intra^c^ | 0.93 (-0.39,2.24) | 1.21  (0.17,2.25) | 3.04  (0.88,5.20) | 0.58  (-0.95,2.11) | 0.93  (0.22,1.65) | -1.84  (-3.43,-0.24) | -0.15  (-1.80,1.50) | -0.19  (-1.03,0.64) | -0.01  (-0.39,0.37) |
|  | Inter^d^ | 0.22 (-1.45,1.89) | 1.73  (-0.14,3.60) | 2.04  (-0.23,4.32) | 0.58  (-1.10,2.27) | 0.35  (-0.77,1.47) | -0.49  (-2.59,1.61) | 1.18  (-1.30,3.66) | 0.20  (-1.06,1.45) | 0.33  (-0.20,0.86) |
| Handgrip strength  (per 6 kg)^b^ | Unadjusted | 0.14* (0.02,0.26) | 0.48*  (0.37,0.60) | 0.35*  (0.19,0.50) | 0.13*  (0.01,0.26) | 0.20*  (0.12,0.28) | -0.27*  (-0.41,-0.12) | -0.24*  (-0.41,-0.07) | -0.14*  (-0.22,-0.06) | -0.17  (-0.38,0.03) |
|  | Adjusted^ab^ | 1.76* (0.52,2.99) | 1.53*  (0.42,2.65) | 3.99*  (2.14,5.83) | 1.99*  (0.64,3.35) | 1.16*  (0.42,1.91) | -2.36*  (-3.90,-0.82) | -1.87*  (-3.53,-0.21) | -1.13*  (-1.98,-0.28) | -0.22  (-0.59,0.15) |
|  | Intra^c^ | 2.27* (0.66,3.88) | 1.09  (-0.21,2.38) | 4.45*  (1.80,7.10) | 2.27*  (0.38,4.15) | 1.47*  (0.58,2.35) | -2.32*  (-4.29,-0.35) | -1.13  (-3.14,0.89) | -0.93  (-1.96,0.10) | -0.08  (-0.54,0.39) |
|  | Inter^d^ | 1.29 (-0.63,3.21) | 2.79*  (0.64,4.95) | 4.25*  (1.65,6.86) | 1.70  (-0.24,3.65) | 1.04  (-0.26,2.33) | -2.43  (-4.87,0.01) | -3.39*  (-6.27,-0.51) | -1.55*  (-3.01,-0.08) | -0.47  (-1.09,14) |

Abbreviations: EORTC QLQ-C30, European Organization for the Research and Treatment of Cancer Quality of Life; β, beta-coefficient; CI, confidence interval; Qol, Quality of life; BMI, body mass index, MUAMC, mid upper arm muscle circumference; cm, centimeter; mm, millimeter; kg, kilogram.

^a^ Model adjusted for sex (male/female), age enrollment (years), co-morbidities (0, 1, ≥2), weeks since end of treatment (weeks), chemotherapy (yes/no), radiotherapy (yes/no), MVPA (hours/weeks), sedentary time (hours/day in bouts per minute), diet quality (range: 0-5), body composition at diagnosis (kg/m² or cm or % or mm or kg), stoma (yes/no), depression (HADS score), smoking (current/former/never), and partner (yes/no).

^b^ The beta-coefficients represent the overall longitudinal difference in the outcome score per 0.5 standard deviation difference in BMI (2.35 kg/m²), waist circumference (6.8 cm), fat percentage (3.2%), MUAMC (14.8 mm), and handgrip strength (6 kg).

^c^ The beta-coefficients represent the change in the outcome score over time within individuals per 0.5 standard deviation increase in BMI (2.35 kg/m²), waist circumference (6.8 cm), fat percentage (3.2%), MUAMC (14.8 mm), and handgrip strength (6 kg).

^d^ The beta-coefficients represent the difference in the outcome score between individuals, per 0.5 standard deviation difference in BMI (2.35 kg/m²), waist circumference (6.8 cm), fat percentage (3.2%), MUAMC (14.8 mm), and handgrip strength (6 kg).

^e^ A random slope was added to the model for BMI with Global QoL, physical functioning, and subjective fatigue; waist circumference with global QoL, role functioning and subjective fatigue; fat percentage with total fatigue, and subjective fatigue; MUAMC with total fatigue, and subjective fatigue; and handgrip strength with global QoL, role functioning, and the summary score (see Methods).
