# Supplementary material for: Increases in adipose tissue and muscle function are longitudinally associated with better quality of life in colorectal cancer survivors
Source: Sci Rep. 2021 Jun 14;11:12440. doi: 10.1038/s41598-021-91709-y (PMC8203762; doi:10.1038/s41598-021-91709-y)
Supplement: Supplementary file 2 — Supplementary Table 2. [file 41598_2021_91709_MOESM2_ESM.docx]

Supplement table 2 Linear mixed models of the five body composition measures (BMI, waist circumference, fat percentage, MUAMC and handgrip strength) in relation to CIPN in participants treated with chemotherapy

|  |  | EORTC QLQ-CIPN20 | | | | | | | |
| --- | --- | --- | --- | --- | --- | --- | --- | --- | --- |
|  | SumScore | | Motoric | | Sensoric | | Autonomic | |  |
|  | β (95% CI) | | β (95% CI) | | β (95% CI) | | β (95% CI) | |  |
| BMI  (per 2.35 kg/m²)^b^ | Unadjusted | 1.35* | (0.41,2.29) | 1.23* | (0.28,2.18) | 1.72* | (0.50,2.94) | 0.58 | (-0.26,1.42) |
|  | Adjusted^ab^ | 0.64 | (-1.46,2.75) | -0.80 | (-3.08,1.47) | 1.62 | (-1.18,4.41) | 0.20 | (-2.02,2.41) |
|  | Intra^c^ | 1.65 | (-0.81,4.11) | 0.35 | (-2.45,3.16) | 2.70 | (-0.57,5.97) | 1.85 | (-1.04,4.73) |
|  | Inter^d^ | -2.24 | (-6.45,1.96) | -3.16 | (-7.21,0.90) | -1.50 | (-7.10,4.11) | -2.33 | (-5.91,1.25) |
| Waist circumference (per 6.8 cm)^b^ | Unadjusted | 0.96* | (0.05,1.88) | 0.63 | (-0.31,1.57) | 1.42* | (0.23,2.60) | 0.57 | (-0.25,1.39) |
|  | Adjusted^ab^ | 0.45 | (-1.36,2.26) | -0.37 | (-2.31,1.58) | 1.15 | (-1.25,3.54) | -0.40 | (-2.26,1.46) |
|  | Intra^c^ | 1.08 | (-1.12,3.28) | 0.18 | (-2.33,2.69) | 1.80 | (-1.11,4.71) | 1.47 | (-1.11,4.06) |
|  | Inter^d^ | -0.97 | (-4.31,2.37) | -1.27 | (-4.51,1.97) | -0.33 | (-4.78,4.12) | -2.89* | (-5.71,-0.07) |
| Fat percentage (per 3.2 %)^b^ | Unadjusted | 0.01 | (-0.87,0.88) | 0.37 | (-0.54,1.27) | -0.18 | (-1.32,0.95) | 0.27 | (-0.52,1.07) |
|  | Adjusted^ab^ | -0.71 | (-2.26,0.84) | -1.38 | (-3.05,0.29) | -0.36 | (-2.42,1.71) | -0.24 | (-1.89,1.40) |
|  | Intra^c^ | -0.39 | (-2.17,1.39) | -1.02 | (-3.04,0.99) | -0.18 | (-2.55,2.18) | 1.46 | (-0.62,3.54) |
|  | Inter^d^ | -1.72 | (-4.91,1.47) | -2.34 | (-5.41,0.74) | -0.92 | (-5.17,3.33) | -3.10* | (-5.75,-0.44) |
| MUAMC  (per 14.8 mm)^b^ | Unadjusted | *0.05* | *(-0.79,0.90)* | *0.12* | *(-0.76,1.00)* | *0.12* | *(-0.98,1.21)* | *0.32* | *(-0.46,1.09)* |
|  | Adjusted^ab^ | -0.32 | (-1.79,1.14) | 0.42 | (-1.22,2.06) | -0.79 | (-2.74,1.16) | -0.26 | (-1.81,1.29) |
|  | Intra^c^ | -0.69 | (-2.38,1.00) | 0.11 | (-1.82,2.03) | -1.51 | (-3.75,0.74) | 0.22 | (-1.77,2.21) |
|  | Inter^d^ | 0.75 | (-2.12,3.62) | 0.40 | (-2.31,3.11) | 1.35 | (-2.49,5.18) | -0.98 | (-3.41,1.45) |
| Handgrip strength  (per 6 kg)^b^ | Unadjusted | -1.19* | (-2.12,-0.25) | -1.85* | (-2.80,-0.91) | -0.67 | (-1.88,0.54) | -0.58 | (-1.43,0.26) |
|  | Adjusted^ab^ | -3.32* | (-4.90,-1.74) | -3.81* | (-5.52,-2.09) | -3.47* | (-5.59,-1.35) | -1.67 | (-3.37,0.03) |
|  | Intra^c^ | -2.76* | (-4.57,-0.94) | -2.93* | (-5.01,-0.86) | -2.96* | (-5.39,-0.52) | -1.04 | (-3.20,1.11) |
|  | Inter^d^ | -5.01* | (-8.13,-1.90) | -5.64* | (-8.61,-2.67) | -5.05* | (-9.26,-0.83) | -2.69 | (-5.41,0.04) |

Abbreviations: EORTC QLQ-CIPN20, European Organization for the Research and Treatment of Cancer Quality of Life Chemotherapy-Induced Peripheral Neuropathy; β, beta-coefficient; CI, confidence interval; BMI, body mass index, MUAMC, mid upper arm muscle circumference; cm, centimeter; mm, millimeter; kg, kilogram.

^a^ Model adjusted for sex (male/female), age enrollment (years), co-morbidities (0, 1, ≥2), weeks since end of treatment (weeks), radiotherapy (yes/no), MVPA (hours/weeks), sedentary time (hours/day in bouts per minute), diet quality (range: 0-5), body composition at diagnosis (kg/m² or cm or % or mm or kg), stoma (yes/no), depression (HADS score), smoking (current/former/never), and partner (yes/no).

^b^ The beta-coefficients represent the overall longitudinal difference in the outcome score per 0.5 standard deviation difference in BMI (2.35 kg/m²), waist circumference (6.8 cm), fat percentage (3.2%), MUAMC (14.8 mm), and handgrip strength (6 kg).

^c^ The beta-coefficients represent the change in the outcome score over time within individuals per 0.5 standard deviation increase in BMI (2.35 kg/m²), waist circumference (6.8 cm), fat percentage (3.2%), MUAMC (14.8 mm), and handgrip strength (6 kg).

^d^ The beta-coefficients represent the difference in the outcome score between individuals, per 0.5 standard deviation difference in BMI (2.35 kg/m²), waist circumference (6.8 cm), fat percentage (3.2%), MUAMC (14.8 mm), and handgrip strength (6 kg).

^e^ A random slope was added to the model for everyone for body fat with autonomic subscale, for MUAMC with summary score, motor subscale and autonomic subscale, and for handgrip strength with summary score, motor subscale, sensory subscale and autonomic subscale. In the model for only chemotherapy patients a random slope was added for waist circumference with autonomic subscale, for body fat with motor subscale, and MUAMC with motor subscale (see Methods).
